# DNA methylation and mRNA expression of glutathione S-transferase alpha 4 are associated with intracranial aneurysms in a gender-dependent manner

**DOI:** 10.3389/fgene.2022.1079455

**Published:** 2023-01-09

**Authors:** Tianqi Xu, Xi Yu, Shenjun Zhou, Yiwen Wu, Xinpeng Deng, Yuefei Wu, Shiyi Wang, Xiang Gao, Sheng Nie, Chenhui Zhou, Jie Sun, Yi Huang

**Affiliations:** ^1^ Department of Neurology, Ningbo First Hospital, Ningbo University, Ningbo, Zhejiang, China; ^2^ Key Laboratory of Precision Medicine for Atherosclerotic Diseases of Zhejiang Province, Ningbo, Zhejiang, China; ^3^ Department of Hepatopancreatobiliary Surgery, Ningbo Medical Center Lihuili Hospital, Ningbo University, Ningbo, Zhejiang, China; ^4^ Department of Neurosurgery, Ningbo First Hospital, Ningbo University, Ningbo, Zhejiang, China; ^5^ Medical School of Ningbo University, Ningbo, China

**Keywords:** intracranial aneurysm, DNA methylation, mRNA expression, 5-aza-2'-deoxycytidine, glutathione S-tranferase alpha 4

## Abstract

**Objective:** We performed a case-control study to investigate the correlation between DNA methylation and mRNA expression of the glutathione S-transferase alpha 4 (*GSTA4*) gene and the risk of intracranial aneurysm (IA) in the Chinese Han population.

**Methods:** After propensity score matching, 44 pairs of cases and controls were collected in this study. Fasting blood samples were collected for DNA and RNA extraction within 24 h of admission. Nine CpG dinucleotides were selected from the GSTA4 promoter region for DNA methylation pyrosequencing. mRNA expression of GSTA4 was measured by quantitative real-time polymerase chain reaction (RT-qPCR). *In vitro* cell experiments were conducted to verify the association between 5-aza-2′-deoxycytidine induced DNA hypomethylation and GSTA4 mRNA expression.

**Results:** The mean methylation level of GSTA4 was much lower in IA patients, especially in IA patients, especially in unruptured IA (UIA), than that in controls (IA vs. Control, *p* < .001; ruptured IA (RIA) vs. Control, *p* = .005; UIA vs. Control, *p* < .001). With sex stratification, we further found that the association between GSTA4 methylation and IA risk presented only in women (mean methylation level: IA vs. Control, *p* < .001; RIA vs. Control, *p* = .009; UIA vs. Control, *p* < .001). GSTA4 mRNA expression was significantly higher in the IA group than in the control group (*p* < .01) and negatively correlated with DNA methylation in all individuals (r = −.746, *p* < .001). DNA hypomethylation can increase GSTA4 mRNA expression in human primary artery smooth muscle cells. The receiver operating characteristic (ROC) curve showed that GSTA4 mean methylation (AUC = .80, *p* < .001) was a reliable predictor of women intracranial aneurysm, among which CpG 1 exhibited the best predictive value (AUC = .89, *p* < .001). In addition, GSTA4 expression levels could also predict the risk of IA in women (AUC = .87, *p* = .005).

**Conclusion:** Decreased DNA methylation and increased mRNA expression of the GSTA4 gene are associated with the risk of IA in women.

## Introduction

Intracranial aneurysm (IA) is a common cerebrovascular disorder that presents as abnormal enlargement of the local cerebral arterial cavity. Rupture of the IA can develop into subarachnoid hemorrhage (SAH), which is a fatal acute event (Etminan and Rinkel, 2016). Epidemiological surveys have shown that IA occurs in one in 20–30 adults, and one in four IAs will rupture during the patient’s lifetime (Korja and Kaprio, 2016; Chen et al., 2018). Congenital cerebral vascular dysplasia is a vital cause of IA. Acquired risk factors, including atherosclerosis, infection, and trauma, aggravate the pathological process (Jin et al., 2019; Etminan et al., 2020). Genome-wide association studies (GWAS) have confirmed that many genes are involved in the pathophysiology of IA (Bakker et al., 2020; Bakker and Ruigrok, 2021). However, the molecular mechanisms underlying IA formation are not yet fully understood.

DNA methylation is the main form of epigenetic modification that can alter gene expression without changing the DNA sequence. DNA methylation in gene promoter regions is closely related to reduced mRNA expression and protein levels (Palou-Marquez et al., 2021). DNA methylation can serve as a bridge between genetic and environmental factors in cerebrovascular diseases (Yu et al., 2017; Law and Holland, 2019). Wang et al. reported that long-term cigarette smoking could increase DNA methylation levels of gene-encoding polypyrimidine tract-binding protein 1 (*PTBP1*), which further reduces PTBP1 expression and contributes to the formation of IA (Wang et al., 2021). Sex plays a vital role in DNA methylation. Liu et al. (2020) observed significant associations between phospholipase A2 group VII (*PLA2G7*) methylation and the risk of developing IA in women.

Literature data showed that the glutathione system might contribute to the formation of aneurysms through mechanisms related to increased oxidative stress, and a variety of glutathione S-transferases (GSTs) may play a role in protecting the cell from oxidative damage (Jeong et al., 2021; Perez-Torres et al., 2022). Glutathione S-transferase alpha 4 (GSTA4) is a phase 2 detoxifying enzyme, a member of the α-class of GSTs (Carlstrom et al., 2020). By catalyzing conjugation reactions of metabolites of lipid peroxidation to glutathione (GSH), GSTA4 participates in regulating cell proliferation and apoptosis (Laborde, 2010). GSTA4 is expressed in the cerebral cortex and brainstem and is involved in the development of gliomas (Cheng et al., 2022). Overexpression of human GSTA4 in carotid arteries can reduce neointimal formation after carotid allografts (Xu et al., 2010). In addition, GSTA4 has been reported to be critical for the phenotypic transition of vascular smooth muscle cells (SMCs). Lou et al. reported that GSTA4 overexpression inhibits SMC proliferation and activation (Luo et al., 2018). SMCs and the collagen fibers they secrete provide structural support to the cerebral arteries. Disruption of the balance between SMC proliferation and apoptosis contributes to IA formation (Frosen, 2014; Kataoka et al., 2020). In this study, we hypothesized that *GSTA4* methylation is involved in the pathological process of IA. In this case-control study, we investigated the association between DNA methylation and mRNA expression of the *GSTA4* gene with the risk of IA in the Chinese Han population.

## Materials and methods

### Participants

This was a single-center case-control study. The clinical sample collection has been described in previously published study (Wang et al., 2021). Briefly, Patients with IA and age-/sex-matched controls were recruited from Ningbo First Hospital. All IA patients were diagnosed using computed tomography angiography (CTA) or digital subtraction angiography (DSA). Healthy controls were excluded if they had cerebrovascular, cardiovascular, or other serious disorders. Fasting blood samples were obtained from participants within 24 h of hospital admission. Plasma levels of triglycerides (TG), total cholesterol (TC), high-density lipoprotein (HDL), and low-density lipoprotein (LDL) were measured using an automatic biochemical analyzer (Olympus AU2700, Tokyo, Japan). After propensity score matching, 44 pairs of cases and controls were included in the following *GSTA4* methylation analysis. There were 22 patients with a ruptured IA (RIA) and 22 with an unruptured IA (UIA) in the case group.

### Pyrosequencing assay

Genomic DNA was extracted from human peripheral blood using an automatic nucleic acid extraction apparatus (LAB-AID 820, Xiamen, China). *GSTA4* is located on Chr6:52,977,953-52,995,284 (GRCh38/hg38). Nine CpG dinucleotides were selected from the promoter region for DNA methylation pyrosequencing. The EpiTect Fast Bisulfite Conversion Kit (Qiagen, Hilden, Germany) was used for bisulfite conversion. DNA methylation levels were measured using PyroMark Q24 (Qiagen). Polymerase chain reaction primers were designed using PyroMark Assay Design software (Qiagen). Primer sequences for the nine CpG regions of *GSTA4* were as follows: forward primer, 5′-AGG​GAA​AAA​AAA​GAA​GAG​AGA​AAG​G-3′; reverse primer, 5′-biotin-TTTAACACTATCCAAAATACCTTACAAA-3′; and sequencing primer, 5′-GGA​GAT​AGA​TTT​GGA​GTT​TA-3′.

### Quantitative real-time polymerase chain reactions (RT-qPCR)

A total of 40 gender-age-matched participants (including 20 IAs and 20 controls) were collected for mRNA detection. The TRIzol-based method was used for DNA-free RNA extraction from blood samples (Meng and Feldman, 2010). The SYBR Green Kit (TaKaRa, Dalian, China) and LightCycler 480 system (Roche, Mannheim, Germany) were used for RT-qPCR. *β-*actin was used as an internal reference, whose primer sequences were described in previous study (Zhang et al., 2018). qPCR primers for *GSTA4* were as follows: forward primer, 5′-GTG​AGA​ACC​GTC​TAC​AAC​AT-30; reverse primer, 5′- TGA​ACT​GGC​TAC​AGG​ACA​T-3′.

### Cell culture and 5-aza-2′-deoxycytidine (AZA) treatment

Human brain vascular smooth muscle cells (HBVSMCs) (Catalog #1100, ScienCell, Carlsbad, Canada) were used in this study. Cells were cultured in six-well plates at a density of 1 × 10^6^ cells/well in smooth muscle cell medium (SMCM) (ScienCell, Carlsbad, Canada). AZA (Sigma-Aldrich; Darmstadt, Germany) is a DNA methyltransferase (DNMT) inhibitor typically used to induce DNA hypomethylation. Cells were treated with three different concentrations of AZA (5, 15, and 25 µM) for 3 days to assess the regulatory role of DNA methylation in *GSTA4* gene expression. Total RNA was extracted from the cells for RT-qPCR analysis.

### Statistical analyses

SPSS (v.23.0; SPSS Inc., Chicago, United States) and GraphPad Prism v.7.0 (GraphPad Software, CA, United States) were used to conduct statistical analyses in this study. Propensity score matching analysis was used to control for confounders on the results. For continuous variables, Student’s *t*-test was used for group comparison. For non-normal continuous variables, the Mann–Whitney *U*-test was used. Associations between *GSTA4* methylation and clinical characteristics were assessed using Pearson or Spearman correlation coefficient analysis. The predictive value of *GSTA4* methylation levels for the risk of IA was evaluated using the receiver operating characteristic (ROC) curve. Statistical significance was set at *p* values <.05.

## Results

**FIGURE 1:**
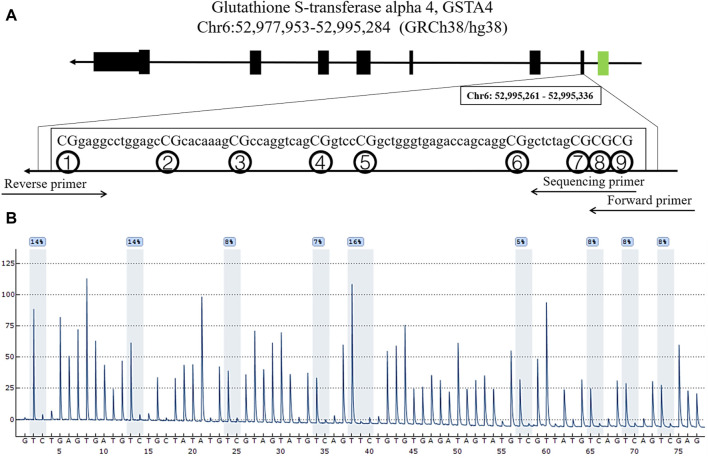
Locations and analysis of nine CpGs in *GSTA4*. **(A)** The locations of the nine CpGs in the *GSTA4* gene. **(B)** Representative sequencing analysis of nine methylation sites.

**FIGURE 2:**
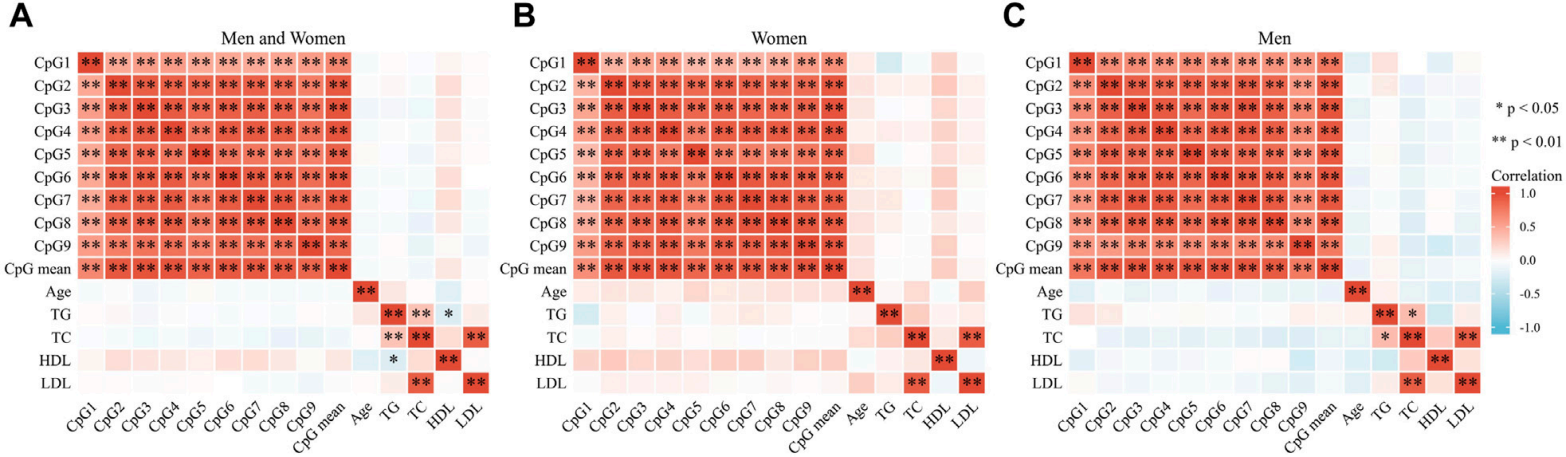
Pearson correlation analysis involving *GSTA4* methylation status and clinical characters. **(A)** In men and women: methylation levels of all nine CpGs were significantly correlated. **(B)** In men: methylation levels of all nine CpGs were significantly correlated. **(C)** In women: methylation levels of all nine CpGs were significantly correlated.

The clinical baseline and other characteristics (such as age, hypertension, diabetes, alcohol drinking, cigarette smoking status, plasma levels of TG, TC, HDL, and LDL, etc.) were not statistically different between IA and control groups (*p* > .05, Table 1). The nine dinucleotide loci (CpG1, CpG2, CpG3, CpG4, CpG5, CpG6, CpG7, CpG8, CpG9) in the *GSTA4* promoter region (Chr6:52,977,953-52,995,284, GRCh38/hg38) were selected for DNA methylation pyrosequencing (Figure 1). Figure 2 shows significant correlations involving the methylation levels of all nine CpGs (*p* < .05). However, age and blood lipids, including TG, TC, LDL, and HDL, were not associated with *GSTA4* methylation in men and women.

**TABLE 1:** The clinical characteristics of participants after propensity score matching.

Character	IA (*n* = 44)	Control (*n* = 44)	t/x	*p*
Age (year)	47.73 ± 5.74	46.30 ± 6.02	1.14	.257
Sex (Male, n)	22	22	.03	.980
Hypertension (n)	8	3	1.22	.206
Diabetes (n)	1	4	.88	.362
Drinking (n)	10	5	1.43	.273
Smoking (n)	12	9	.35	.631
TG (mmol/L)	1.53 ± .87	1.61 ± 1.41	.31	.754
TC (mmol/L)	4.96 ± .81	4.80 ± .88	.89	.372
HDL (mmol/L)	1.19 ± .23	1.27 ± .26	1.48	.140
LDL (mmol/L)	3.05 ± .68	2.94 ± .62	.77	.441

TG, Triglycerides; TC, Total cholesterol; HDL, High-density lipoprotein; LDL, Low-density lipoprotein.

The DNA methylation levels of *GSTA4* in the IAs were lower than those in the controls. As shown in Table 2, the significant differences were found in CpG1 (IA vs. Control, *p* = .001; RIA vs. Control, *p* = .033; UIA vs. Control, *p* = .001), CpG2 (IA vs. Control, *p* = .002; RIA vs. Control, *p* = .035; UIA vs. Control, *p* = .002), CpG3 (IA vs. Control, *p* < .001; UIA vs. Control, *p* = .001; RIA vs. Control, *p* = .001), CpG4 (IA vs. Control, *p* < .001; RIA vs. Control, *p* = .003), CpG5 (IA vs. Control, *p* = .024; RIA vs. Control, *p* = .043; UIA vs. Control, *p* = .001), CpG6 (IA vs. Control, *p* = .001; RIA vs. Control, *p* = .008; UIA vs. Control, *p* < .001), CpG7 (IA vs. Control, *p* = .001; RIA vs. Control, *p* = .023; UIA vs. Control, *p* < .001), CpG8 (IA vs. Control, *p* < .001; RIA vs. Control, *p* = .011; UIA vs. Control, *p* = .029), CpG9 (IA vs. Control, *p* = .034; UIA vs. Control, *p* = .001), Mean methylation levels of the nine CpG sites (IA vs. Control, *p* < .001; RIA vs. Control, *p* = .005; UIA vs. Control, *p* < .001).

**TABLE 2:** DNA methylation differences of nine CpGs dinucleotides of *GSTA4* gene between IAs and controls.

Character	Control (*n* = 44)	IA (*n* = 44)	RIA (*n* = 22)	UIA (*n* = 22)	*p* IA vs. control	*p* RIA vs. control	*p* UIA vs. control
CpG1 (%)	12.84 ± 3.23	10.33 ± 3.55	10.91 ± 3.71	9.75 ± 3.36	**.001**	**.033**	**.001**
CpG2 (%)	10.28 ± 3.73	7.99 ± 2.95	8.26 ± 3.28	7.72 ± 2.64	**.002**	**.035**	**.002**
CpG3 (%)	7.13 ± 2.42	5.49 ± 1.36	5.52 ± 1.26	5.45 ± 1.48	**<.001**	**.001**	**.001**
CpG4 (%)	6.73 ± 2.37	5.09 ± 1.26	5.31 ± 1.41	4.88 ± 1.08	**<.001**	**.003**	.074
CpG5 (%)	11.37 ± 3.51	9.73 ± 3.19	9.81 ± 2.55	9.66 ± 3.79	**.024**	**.043**	**.001**
CpG6 (%)	5.68 ± 1.71	4.55 ± 1.20	4.62 ± 1.31	4.47 ± 1.10	**.001**	**.008**	**<.001**
CpG7 (%)	7.30 ± 2.07	6.04 ± 1.30	6.22 ± 1.59	5.87 ± .92	**.001**	**.023**	**<.001**
CpG8 (%)	7.27 ± 1.80	6.05 ± 1.16	6.30 ± 1.16	5.80 ± 1.13	**<.001**	**.011**	**.029**
CpG9 (%)	6.71 ± 2.02	5.94 ± 1.25	6.10 ± 1.15	5.77 ± 1.34	**.034**	.127	**.001**
Mean (%)	8.37 ± 2.30	6.80 ± 1.52	7.01 ± 1.50	6.60 ± 1.55	**<.001**	**.005**	**<.001**

Abbreviation: GSTA4, Glutathione S-transferase alpha 4; IA, intracranial aneurysm; RIA, ruptured intracranial aneurysm; UIA, unruptured intracranial aneurysm. Data were presented as mean ± SD. The *t*-test was used for group comparison. The *p*-value less than or equal to .05 is in bold.

Sex is a crucial factor that influences DNA methylation levels. With sex stratification, we further found that the association between *GSAT4* methylation and IA risk was present only in women. As shown in Table 3, the significant differences were found in CpG1 levels among women (IA vs. Control, *p* < .001; RIA vs. Control, *p* = .001; UIA vs. Control, *p* < .001), CpG2 (IA vs. Control, *p* = .005; UIA vs. Control, *p* = .006), CpG3 (IA vs. Control, *p* = .001; RIA vs. Control, *p* = .002; UIA vs. Control, *p* = .011), CpG4 (IA vs. Control, *p* < .001; RIA vs. Control, *p* = .020; UIA vs. Control, *p* = .002), CpG5 (IA vs. Control, *p* = .026; RIA vs. Control, *p* = .039), CpG6 (IA vs. Control, *p* = .002; RIA vs. Control, *p* = .039; UIA vs. Control, *p* = .002), CpG7 (IA vs. Control, *p* = .003; UIA vs. Control, *p* < .001), CpG8 (IA vs. Control, *p* = .002; UIA vs. Control, *p* < .001), CpG9 (IA vs. Control, *p* = .001; RIA vs. Control, *p* = .04; UIA vs. Control, *p* = .003), Mean methylation levels of the nine CpG sites (IA vs. Control, *p* < .001; RIA vs. Control, *p* = .009; UIA vs. Control, *p* < .001). However, no significant differences were found in mean methylation level in men (*p* > .05, Table 4). Subsequent sex comparison analysis showed that compared to that in healthy men, healthy women had higher *GSTA4* methylation levels CpG1 (*p* = .004, Table 5), CpG2 (*p* = .024), CpG3 (*p* = .042), CpG4 (*p* = .041), CpG6 (*p* = .038), CpG8 (*p* = .035), CpG9 (*p* = .02), mean methylation levels of the nine CpG sites (*p* = .019). However, only CpG1 presented a significant difference between women and man IA (*p* = .013).

**TABLE 3:** DNA methylation differences of nine CpGs dinucleotides of *GSTA4* gene between IA and control in women.

Character	Control (*n* = 22)	IA (*n* = 22)	RIA (*n* = 11)	UIA (*n* = 11)	*p* IA vs. control	*p* RIA vs. control	*p* UIA vs. control
CpG1 (%)	14.19 ± 3.24	9.03 ± 3.19	9.31 ± 3.91	8.74 ± 2.44	**<.001**	**.001**	**<.001**
CpG2 (%)	11.53 ± 3.65	8.31 ± 3.54	8.83 ± 4.07	7.78 ± 3.04	**.005**	.063	**.006**
CpG3 (%)	7.86 ± 2.35	5.71 ± 1.49	5.70 ± 1.39	5.42 ± 1.72	**.001**	**.002**	**.011**
CpG4 (%)	7.46 ± 2.47	5.11 ± 1.35	5.42 ± 1.72	4.80 ± .82	**<.001**	**.020**	**.002**
CpG5 (%)	12.20 ± 3.83	9.69 ± 3.36	9.79 ± 2.52	9.60 ± 4.16	**.026**	**.039**	.084
CpG6 (%)	6.21 ± 1.80	4.61 ± 1.43	4.81 ± 1.68	4.41 ± 1.17	**.002**	**.039**	**.002**
CpG7 (%)	7.83 ± 1.97	6.16 ± 1.59	6.62 ± 1.96	5.70 ± .98	**.003**	.106	**<.001**
CpG8 (%)	7.83 ± 1.77	6.28 ± 1.23	6.69 ± 1.31	5.86 ± 1.02	**.002**	.070	**<.001**
CpG9 (%)	7.41 ± 1.71	5.80 ± 1.34	6.13 ± 1.33	5.47 ± 1.31	**.001**	**.040**	**.003**
Mean (%)	9.17 ± 2.25	6.74 ± 1.55	7.03 ± 1.69	6.45 ± 1.43	**<.001**	**.009**	**<.001**

Abbreviation: GSTA4, Glutathione S-transferase alpha 4; IA, intracranial aneurysm; RIA, ruptured intracranial aneurysm; UIA, unruptured intracranial aneurysm. Data were presented as mean ± SD. The *t*-test was used for group comparison. The *p*-value less than or equal to .05 is in bold.

**TABLE 4:** DNA methylation differences of nine CpGs dinucleotides of *GSTA4* gene between IA and control in men.

Character	Control (*n* = 22)	IA (*n* = 22)	RIA (*n* = 11)	UIA (*n* = 11)	*p* IA vs. control	*p* RIA vs. control	*p* UIA vs. control
CpG1 (%)	11.50 ± 2.66	11.64 ± 3.47	12.51 ± 2.83	10.77 ± 2.94	.879	.322	.536
CpG2 (%)	9.02 ± 3.45	7.68 ± 2.25	7.69 ± 2.31	7.66 ± 2.31	.134	.258	.124
CpG3 (%)	6.39 ± 2.31	5.27 ± 1.22	5.35 ± 1.15	5.20 ± 1.33	.053	.17	.07
CpG4 (%)	6.00 ± 2.08	5.08 ± 1.19	5.19 ± 1.09	4.96 ± 1.33	.078	.152	.141
CpG5 (%)	10.55 ± 3.02	9.78 ± 3.09	9.82 ± 2.71	9.73 ± 3.57	.405	.503	.494
CpG6 (%)	5.15 ± 1.47	4.48 ± .94	4.44 ± .85	4.53 ± 1.06	.083	.151	.224
CpG7 (%)	6.77 ± 2.07	5.93 ± .95	5.82 ± 1.05	6.03 ± .86	.093	.09	.161
CpG8 (%)	6.70 ± 1.67	5.83 ± 1.06	5.91 ± .86	5.74 ± 1.27	**.046**	.083	.104
CpG9 (%)	6.00 ± 2.10	6.07 ± 1.16	6.07 ± .99	6.07 ± 1.37	.903	.928	.927
Mean (%)	7.57 ± 2.11	6.86 ± 1.53	6.98 ± 1.37	6.74 ± 1.73	.211	.409	.273

Abbreviation: GSTA4, Glutathione S-transferase alpha 4; IA, intracranial aneurysm. Data were presented as mean ± SD. The *t*-test was used for group comparison. The *p*-value less than or equal to .05 is in bold.

**TABLE 5:** The gender differences in DNA methylation of nine CpGs of *GSTA4* gene in IAs and controls respectively.

Character	Men	Women	t	p
All	(*n* = 44)	(*n* = 44)		
CpG1 (%)	11.57 ± 3.06	11.61 ± 4.11	.05	.962
CpG2 (%)	8.35 ± 2.96	9.92 ± 3.91	2.12	**.037**
CpG3 (%)	5.83 ± 1.91	6.78 ± 2.23	2.15	**.034**
CpG4 (%)	5.54 ± 1.74	6.29 ± 2.30	1.72	.090
CpG5 (%)	10.16 ± 3.05	10.95 ± 3.78	1.07	.287
CpG6 (%)	4.82 ± 1.26	5.41 ± 1.80	1.79	.078
CpG7 (%)	6.35 ± 1.65	6.99 ± 1.96	1.66	.100
CpG8 (%)	6.26 ± 1.45	7.05 ± 1.70	2.35	**.021**
CpG9 (%)	6.04 ± 1.68	6.60 ± 1.72	1.56	.121
Mean (%)	7.21 ± 1.86	7.96 ± 2.27	1.68	.096
IA	(*n* = 22)	(*n* = 22)		
CpG1 (%)	11.64 ± 3.47	9.03 ± 3.19	2.61	**.013**
CpG2 (%)	7.68 ± 2.25	8.31 ± 3.55	.7	.487
CpG3 (%)	5.27 ± 1.22	5.71 ± 1.49	1.06	.297
CpG4 (%)	5.07 ± 1.19	5.11 ± 1.35	.09	.925
CpG5 (%)	9.78 ± 3.09	9.69 ± 3.36	.08	.935
CpG6 (%)	4.48 ± .94	4.61 ± 1.43	.34	.735
CpG7 (%)	5.93 ± .95	6.16 ± 1.59	.58	.561
CpG8 (%)	5.83 ± 1.06	6.28 ± 1.23	1.31	.199
CpG9 (%)	6.07 ± 1.16	5.80 ± 1.34	.702	.487
Mean (%)	6.86 ± 1.53	6.74 ± 1.55	.25	.803
Control	(*n* = 22)	(*n* = 22)		
CpG1 (%)	11.50 ± 2.66	14.19 ± 3.24	3	**.004**
CpG2 (%)	9.02 ± 3.45	11.53 ± 3.65	2.35	**.024**
CpG3 (%)	6.39 ± 2.31	7.86 ± 2.35	2.1	**.042**
CpG4 (%)	6.00 ± 2.08	7.46 ± 2.47	2.11	**.041**
CpG5 (%)	10.55 ± 3.02	12.20 ± 3.83	1.59	.121
CpG6 (%)	5.15 ± 1.47	6.21 ± 1.79	2.14	**.038**
CpG7 (%)	6.77 ± 2.08	7.83 ± 1.97	1.73	.091
CpG8 (%)	6.70 ± 1.67	7.83 ± 1.77	2.18	**.035**
CpG9 (%)	6.01 ± 2.10	7.41 ± 1.71	2.42	**.020**
Mean (%)	7.57 ± 2.11	9.17 ± 2.25	2.44	**.019**

Abbreviation: GSTA4, Glutathione S-transferase alpha 4; IA, intracranial aneurysm; Data were presented as mean ± SD. The *t*-test was used for group comparison. The *p*-value less than or equal to .05 is in bold.

**FIGURE 3:**
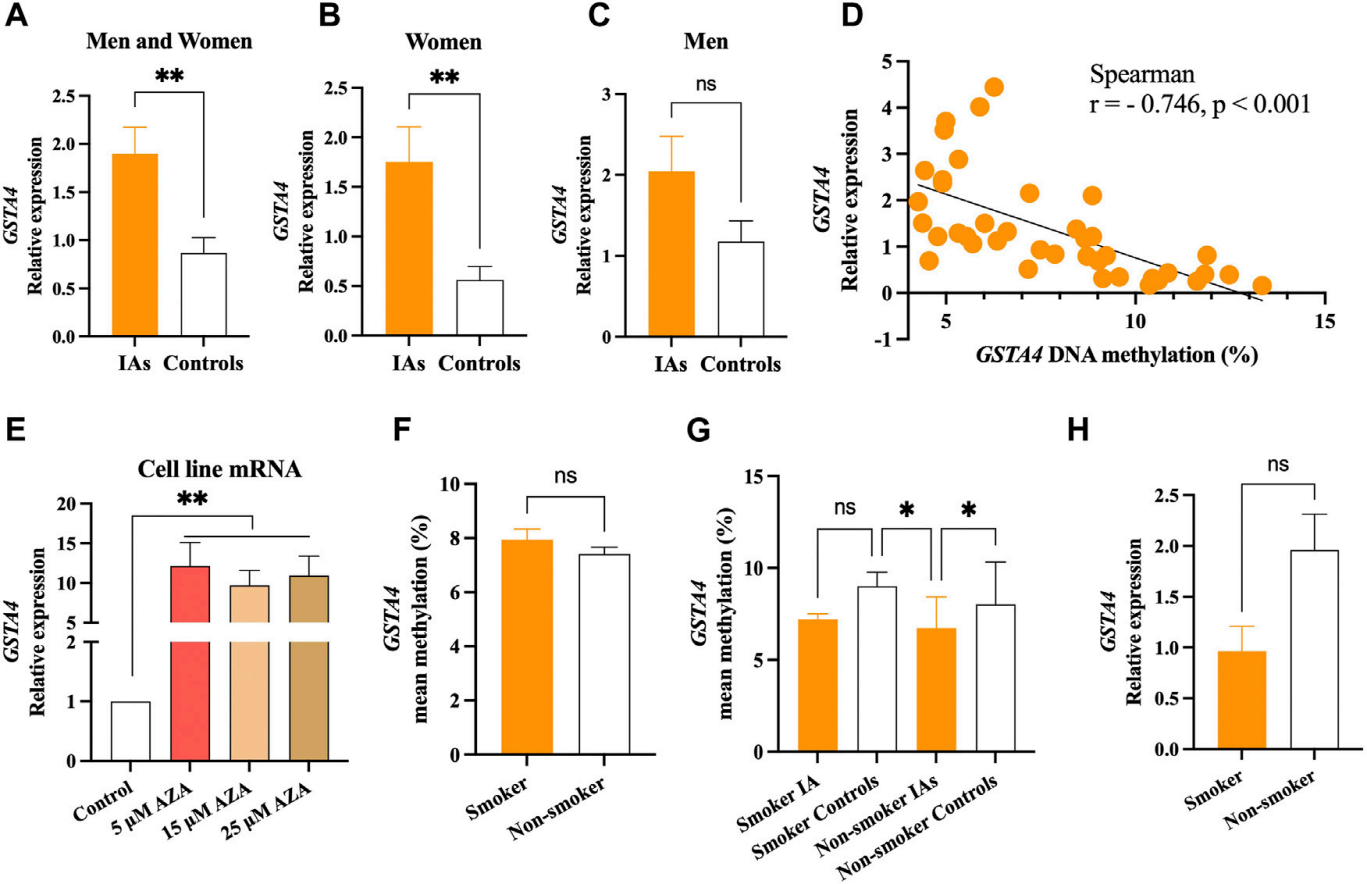
Significant association between *GSTA4* mRNA expression and DNA methylation. **(A)** mRNA expression levels in the IA group were higher than that in controls. **(B)** mRNA expression levels in women IAs were higher than that in women controls. **(C)** No statistical significance was observed between men IAs and men controls. **(D)** *GSTA4* mRNA expression was correlated with its DNA methylation levels in all participants. **(E)** DNA hypomethylation induced by AZA increased the mRNA expression of the *GSTA4* gene in HBVSMCs. **(F,G)** *GSTA4* methylation levels in between smoker and non-smoker group. **(H)** *GSTA4* mRNA expression between smoker and non-smoker groups.

*GSTA4* mRNA expression was significantly higher in the IA group than in the control group (*p* < .01, Figure 3A). Sex group analysis showed that *GSTA4* mRNA expression was significantly higher in the women IA group (*p* < .01, Figure 3B), but not in men IA group (*p* > .05, Figure 3C). In addition, spearman correlation analysis indicated that *GSTA4* mRNA expression was negatively correlated with DNA methylation levels in all individuals (r = −.746, *p* < .001, Figure 3D). *In vitro* experiments also demonstrated that DNA hypomethylation induced by AZA could increase *GSTA4* mRNA expression in HBVSMCs (*p* < .01, Figure 3E).

It is well known that cigarette smoking influences DNA methylation levels, contributing to the formation of IA. Therefore, we performed subgroup analysis stratified by smoker or non-smoker in all participants. No significant differences were found in *GSTA4* methylation between smoker and non-smoker group (*p* > .05, Figure 3F). The *GSTA4* methylation in non-smoker IAs were much lower than that in the smoker controls and non-smoker controls (*p* < .05), but no statistical differences were found in smoker IAs (*p* > .05, Figure 3G). A similar result was also shown in *GSTA4* mRNA expression between smoker and non-smoker groups (*p* > .05, Figure 3H).

**FIGURE 4:**
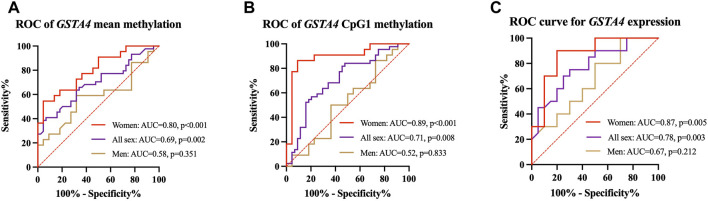
ROC curves for prediction of IAs in men and women. **(A)** *GSTA4* mean methylation is a predictor of women IA. **(B)** *GSTA4* CpG1 methylation is a predictor of women IA. **(C)** *GSTA4* expression is a predictor of women IA.

With subgroup comparisons, we discovered that decreased DNA methylation and increased mRNA expression of *GSTA4* were associated with the risk of IA in women but not in men. The predictive power of *GSTA4* methylation status on IA was further tested using ROC curve analysis. We found that *GSTA4* mean methylation (AUC = .80, *p* < .001, Figure 4A) was a reliable predictor of women IA, of which CpG1 exhibited the best predictive value (AUC = .89, *p* < .001, Figure 4B). In addition, *GSTA4* expression levels could also predict the risk of IA in women (AUC = .87, *p* = .005, Figure 4C).

## Discussion

This case-control study investigated the association between *GSTA4* methylation and expression and IA risk. We found that DNA methylation of *GSTA4* negatively correlated with its mRNA expression. Patients exhibited decreased methylation and increased expression of *GSTA4* compared to that in controls. *GSTA4* methylation and expression are reliable predictors of IA in women.

GSTA4 is a phase 2 detoxifying enzyme that modulates cell proliferation and apoptosis by regulating lipid peroxidation. Previous studies have reported that decreased expression of GSTA4 is associated with positive outcomes in glioma models (Cheng et al., 2022), whereas increased expression of GSTA4 promotes the malignant progression of liver cancer (Liu et al., 2017). Notably, GSTA4 is involved in SMC proliferation (Luo et al., 2018) and may thus play a vital role in the pathological process of IA. As a member of the human α-class of GSTs, *GSTA4* is the only one that has a gene promoter CpG island (Peng et al., 2008). Promoter DNA methylation usually downregulates mRNA levels and, consequently, protein expression of target genes (Moore et al., 2013). Our data also indicated that decreased *GSTA4* promoter methylation is associated with increased *GSTA4* mRNA expression.

The main finding of this study was that patients have lower *GSTA4* methylation and higher *GSTA4* expression than that in healthy controls. The pathological formation of IA is influenced by complex interactions between genetic and environmental risk factors such as congenital dysgenesis and acquired atherosclerosis of the cerebral arteries (Lawton et al., 2015; Zhong et al., 2021). A previous study reported that overexpressing GSTA4 can prevent neointima formation in carotid arteries and suppress SMC activation and proliferation (Xu et al., 2010; Luo et al., 2018). SMCs and the collagen fibers they secrete provide structural support to cerebral arteries. Disruption of the balance between SMC proliferation and apoptosis potentially contributes to IA formation (Frosen, 2014; Kataoka et al., 2020). This may partly explain why patients with IA tended to have lower *GSTA4* methylation and higher *GSTA4* expression. In addition, as an epigenetic modification, DNA methylation can be involved in disease progression and serve as a marker of environmental exposures, such as tobacco smoking, lipid peroxidation, and inflammation (Gao et al., 2019; Christiansen et al., 2021). Consistent with this hypothesis, DNA methylation of the *GSTA4* promoter, which contains antioxidant response elements, may act as an adaptive mechanism through which they are transcriptionally activated during exposure to lipid peroxidation of the vascular wall in IA patients (Benes et al., 2013). Therefore, lower levels of *GSTA4* methylation may serve as a biomarker of vascular impairment in patients with IA.

There are sex-based differences in morbidity, rupture risk, and prognosis in patients (Korja et al., 2014; Hu et al., 2019; Ogilvy et al., 2020). DNA methylation reportedly plays a vital role in sex-specific characteristics in the human brain (Xu et al., 2014; Xia et al., 2021). In addition, GSTA4 expression has sexually differential characteristics that are probably regulated by female hormones (Faustino et al., 2012; Tierbach et al., 2018). In this study, we observed that healthy women had higher *GSTA4* methylation levels than healthy men. Notably, *GSTA4* methylation and expression are reliable predictors of IA risk in women but not in men. Recent studies have reported that GSTA4 knockout results in more severe hearing loss in female mice than in male mice in a cisplatin toxicity experiment (Park et al., 2019). Collectively, these results suggest that interactions between estrogen and *GSTA4* methylation may affect IA progression.

Our study had certain limitations. First, *GSTA4* methylation levels in UIA patients were slightly lower than those in RIA patients. However, this was not a statistically significant difference, which may be attributed to the relatively small sample size. Second, our results showed that *GSTA4* methylation was not significantly associated with smoking, which may be due to the fact that too few smoking patients were included in the analysis. Therefore, external validation trials with larger cohorts are essential. Third, *in vivo* animal experiments are needed to verify the effects of *GSTA4* overexpression or suppression on the SMC phenotypic transition and IA formation. In future studies, we will further supplement this finding.

In conclusion, we established that IA patients had lower levels of *GSTA4* promoter methylation and higher *GSTA4* expression than controls. *GSTA4* methylation and mRNA expression may be reliable predictive markers of the occurrence of women IA.

## Data Availability

The original contributions presented in the study are included in the article/Supplementary Materials, further inquiries can be directed to the corresponding authors.
